# Compassionate care in a digital world: Artificial intelligence and the consultation

**DOI:** 10.4102/phcfm.v18i1.5531

**Published:** 2026-05-18

**Authors:** Werner Viljoen

**Affiliations:** 1 Division of Family Medicine and Primary Care, Faculty of Medicine and Health Sciences, Stellenbosch University, Cape Town, South Africa; 2 Department of Health and Wellness, Helderberg Hospital, Western Cape Government, Cape Town, South Africa

The theme of World Family Doctor Day in 2026 is ‘compassionate care in a digital world’. Dr Viviana Martinez-Bianchi, in her inaugural address as president of the World Organization of Family Doctors, called on family doctors ‘to engage with digital health and artificial intelligence in ways that protect equity and strengthen relationships’.

Some patients are already using artificial intelligence (AI) and more will. They paste laboratory results into a chat window. They describe symptoms that have been worrying them for weeks. They ask about medications, diagnoses, and frightening possibilities. Sometimes they ask questions they may never ask us directly. By the time some patients arrive in the consultation room, they have already had a long conversation with a machine. Something fundamental in medicine is shifting.

Traditionally, when taking a clinical history, the consultation began with an asymmetry of knowledge. The patient came with symptoms. The physician came with training, experience, and access to medical knowledge that was largely out of reach for the public. Part of our role was translation – turning specialised knowledge into something understandable and useful. This asymmetry is dissolving.

When primary care carries the weight of entire health systems, this shift may matter even more. Anyone with a smartphone and a data connection can now access systems capable of synthesising large amounts of medical information. The instinctive reaction among clinicians is often unease. It can feel as if the ground beneath the traditional consultation is shifting. But something more interesting is happening.

Information is becoming abundant, but wisdom remains scarce. Family medicine has never been defined by possessing information alone. At its heart, the discipline is about *care in context*. A patient does not present as a diagnostic puzzle waiting to be solved. They arrive carrying the realities of their lives – work, family, culture, poverty, grief, resilience, and hope. The task of the family physician is to hold those realities together with clinical reasoning and arrive at decisions that are medically sound and *humanly appropriate*. Artificial intelligence can generate possibilities, but it cannot hold a person’s life in view. It can organise information, but only humans can hold meaning.

Consider a familiar consultation in African primary care settings: a patient presenting with cough. Tuberculosis is an obvious clinical possibility. But the consultation rarely stops there. The story often includes overcrowded housing, limited transport to clinics, fear of stigma, and the economic consequences of missing work for treatment. These contextual realities determine whether any management plan succeeds or fails. No algorithm, however sophisticated, can fully grasp lived complexity.

Compassionate care therefore remains irreducibly human. Yet AI does have a key role to play in strengthening clinical practice. Used thoughtfully, it can assist clinicians in synthesising new evidence, reviewing medication interactions, summarising guidelines, and structuring documentation. These tasks increasingly dominate clinical work. When supported by AI tools, physicians may regain something that has been slowly eroded by administrative complexity. As Topol argues, the convergence of human and AI may ultimately allow clinicians to focus more fully on the human dimensions of care.^1^

Time. Time to listen more carefully. Time to ask the second question. Time to notice what the patient is struggling to say. In this sense, the ethical promise of AI in healthcare is not that it will make medicine more technological, but that it may allow medicine to become more human again.

At the same time, the integration of AI into African health systems raises important ethical questions. Many clinical algorithms and large language models are trained predominantly on datasets derived from high-income countries. Evidence already shows that such systems can reproduce and amplify existing biases within healthcare.^2^ Applying these technologies uncritically in different epidemiological and social contexts risks clinical misrepresentation and inequitable care. There are also questions of data colonialism, where data generated in low- and middle-income settings may be extracted to train technologies that primarily benefit other health systems.^3^ Ensuring that AI strengthens rather than distorts primary care in Africa will require careful governance, local participation in model development, and continued emphasis on contextual clinical judgement.^4^

The consultation itself may also begin to change. Instead of starting with diagnostic uncertainty, the conversation may increasingly begin with a patient saying: ‘I asked an AI system, and it suggested these possibilities. What do you think?’ That moment is not a threat to clinical practice. It is an invitation to step into.

The role of the family physician shifts slightly. We are no longer the sole gatekeepers of information. Instead, we become interpreters within a landscape where information is widely accessible but often poorly contextualised or misunderstood. When patients bring AI-generated possibilities, our task is to explore them together – identifying what is plausible, what is unlikely, and how those possibilities interact with the patient’s particular circumstances.

Family medicine is uniquely equipped for this role. Across Africa, primary care has always required clinicians to navigate uncertainty, with limited resources and complex social realities. Continuity of care, community orientation, and attention to the social determinants of health are not theoretical ideals here; they are daily practice. These values provide a powerful framework for guiding the ethical integration of AI into African health systems.

The central question is therefore not whether machines will replace physicians. The more important question is whether physicians will use these technologies in ways that strengthen or weaken *the human relationships at the centre of care*.

The future consultation may involve two forms of intelligence on both sides of the desk. Patients may arrive having consulted AI. Physicians may use similar tools to synthesise information and support decision-making. But the centre of the encounter must remain unmistakably human.

A patient does not come to a doctor seeking an algorithm. They come seeking understanding from someone who can see them not simply as a case, but as a person embedded within a family, a community, and a life. Meaning in medicine has never come from information alone. It comes from the relationship between two human beings sitting in a room trying to understand illness together.

## References

[1] Topol E. High-performance medicine: The convergence of human and artificial intelligence. Nat Med. 2019;25(1):44–56. 10.1038/s41591-018-0300-7 30617339

[2] Obermeyer Z, Powers B, Vogeli C, Mullainathan S. Dissecting racial bias in an algorithm used to manage the health of populations. Science. 2019;366(6464):447–453. 10.1126/science.aax2342 31649194

[3] Birhane A. Algorithmic colonization of Africa. SCRIPTed. 2020;17(2):389–409. 10.2966/scrip.170220.389

[4] Morley J, Machado C, Burr C, et al. The ethics of AI in health care: A mapping review. Soc Sci Med. 2020;260:113172. 10.1016/j.socscimed.2020.113172 32702587

